# l-Me­thion­yl-l-tyrosine monohydrate

**DOI:** 10.1107/S2414314623005515

**Published:** 2023-06-30

**Authors:** Sainath Babu, Michelle O. Claville, Frank R. Fronczek, Rao M. Uppu

**Affiliations:** a Department of Biological Science, Hampton University, Hampton, VA 23668, USA; b School of Science, Hampton University, Hampton, VA 23668, USA; c Department of Chemistry, Louisiana State University, Baton Rouge, LA 70803, USA; d Department of Environmental Toxicology, Southern University and A&M College, Baton Rouge, LA 70813, USA; University of Aberdeen, United Kingdom

**Keywords:** crystal structure, zwitterion, oxidation, nitration

## Abstract

The study of the oxidation of various proteins necessitates scrutiny of the amino acid sequence. Since me­thio­nine (Met) and tyrosine (Tyr) are easily oxidized, peptides that contain these amino acids are frequently studied using a variety of oxidation methods, including, but not limited to, pulse radiolysis, electrochemical oxidation, and laser flash photolysis. To date, the oxidation of the Met–Tyr dipeptide is not fully understood. Several investigators have proposed a mechanism of intra­molecular electron transfer between the sulfide radical of Met and the Tyr residue. Our elucidation of the structure and absolute configuration of l-Met–l-Tyr monohydrate, C_14_H_20_N_2_O_4_S·H_2_O (systematic name: (2*S*)-2-{[(2*S*)-2-amino-4-methyl­sulfanyl­butano­yl]amino}-3-(4-hy­droxy­phen­yl)propanoic acid monohydrate) is presented herein and provides information about the zwitterionic nature of the dipeptide. We suspect that the zwitterionic state of the dipeptide and its inter­action within the solvent medium may play a major role in the oxidation of the dipeptide. In the crystal, all the potential donor atoms inter­act *via* strong N—H⋯O, C—H⋯O, O—H⋯S, and O—H⋯O hydrogen bonds.

## Abstract 2

The hydrated title compound is zwitterionic with the amine group of the me­thionyl moiety protonated and the carboxyl group of tyrosine deprotonated.

## Graphical abstract

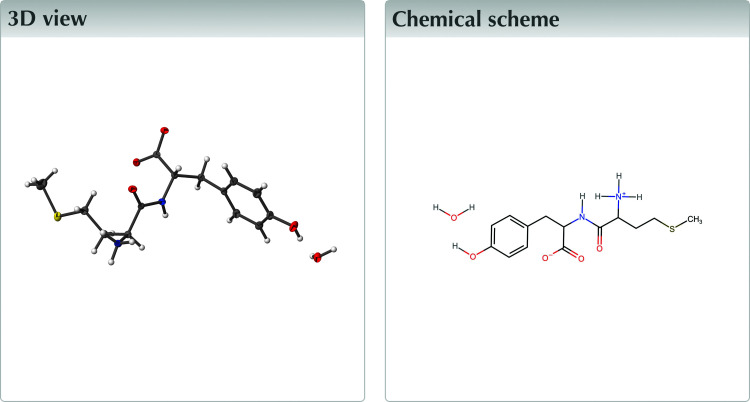

## Structure description

Protein oxidation is an important physiological and pathological mechanism (Berlett & Stadtman, 1997 ▸; Wojcik *et al.*, 2008 ▸). Oxidation of tyrosine (Tyr) and me­thio­nine (Met) residues play a role in the etiology of inflammatory diseases (Gu *et al.*, 2015 ▸; Meredith *et al.*, 2014 ▸). Studies have shown that the Met–Tyr dipeptide has a significant antioxidant activity against the radical cation of 2,2′-azinobis(3-ethylbenzothiazoline-6-sulfonic acid) (ABTS^+**.**^) and peroxyl radicals, while the Tyr–Met dipeptide does not have any reaction with those radicals (Torkova *et al.*, 2015 ▸). The presence of a C-terminal Met group to Tyr had somewhat conflicting results with many oxidation systems (Zhang *et al.*, 2009 ▸; Wojcik *et al.*, 2008 ▸; Nagy *et al.*, 2009 ▸). Several studies have suggested that the mechanism of oxidation is through intramol­ecular electron transfer from Met to Tyr phen­oxy radicals (Bergès *et al.*, 2011 ▸; Houée-Lévin *et al.*, 2015 ▸; Kciuk *et al.*, 2005 ▸; Zhang *et al.*, 2009 ▸). The diverse oxidation ability of the dipeptides could be attributed to the structural differences, particularly the configuration of the zwitterion and their inter­action with solvent mol­ecules. With this in mind, we have elucidated the structure of l-Met–l-Tyr to better understand its role in the oxidation and nitration process.

**Figure 1:**
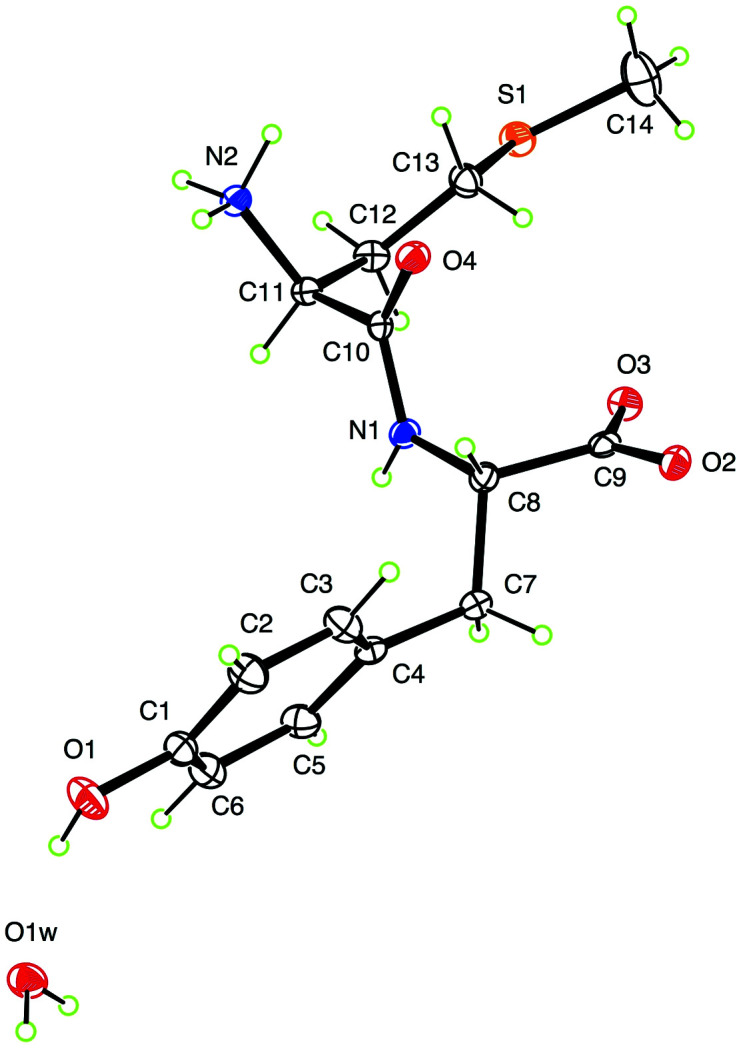
The asymmetric unit of the title compound, shown with 50% probability displacement ellipsoids.

The title compound, l-Met–l-Tyr monohydrate, C_14_H_20_N_2_O_4_S·H_2_O (Fig. 1 ▸), has been analyzed as part of broader studies on the redox properties of Met-containing dipeptides. Within the dipeptide, the amine group of Met is protonated and the carboxyl group of tyrosine is deprotonated, thereby generating a zwitterionic configuration. The conformation of the dipeptide mol­ecule can be qu­anti­fied by four torsion angles. Besides the expected essentially planar peptide linkage, the tyrosine portion has C10—N1—C8—C7 = 165.36 (17)° and N1—C8—C7—C4 = −71.1 (2)°. The me­thio­nine portion has C10—C11—C12—C13 = −45.6 (2)° and the sulfur-containing substituent shows an extended conformation with C11—C12—C13—S1 = −173.31 (13)°.

This structure has been reported recently (Babu *et al.*, 2023 ▸). The title compound (Fig. 1 ▸), derived from two amino acids, l-Met and l-Tyr, crystallized as a monohydrate and was structurally analyzed as a part of broader studies on the redox properties of Met dipeptides. The absolute configuration determined from the X-ray data agrees with that of the starting materials.

**Figure 2:**
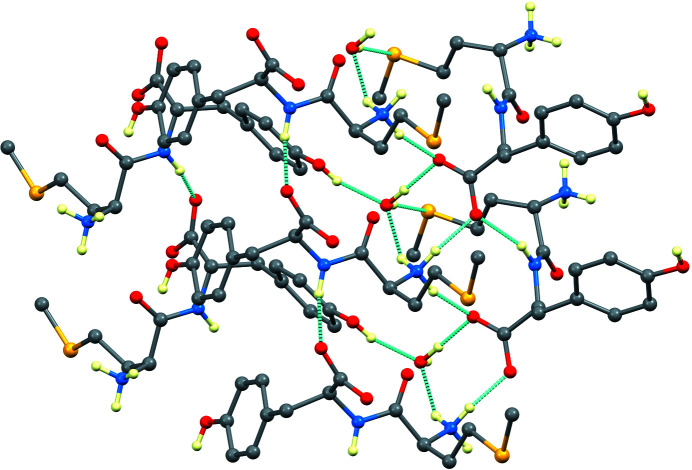
The hydrogen-bonding network, showing only the H atoms involved in hydrogen bonds.

**Figure 3:**
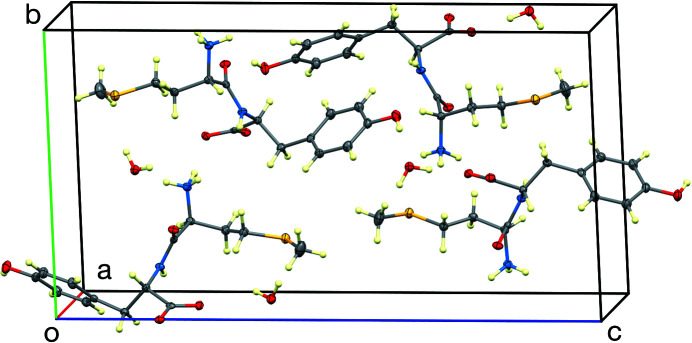
The unit cell, viewed approximately down [100].

In the crystal, the mol­ecules inter­act with each other *via* strong inter­molecular N—H⋯O, C—H⋯O, O—H⋯S, and O—H⋯O hydrogen bonds, forming a three-dimensional network (Table 1 ▸ and Fig. 2 ▸). All the hydrogen-bond donors in the hy­droxy, amidine, and carboxyl­ate groups, as well as the solvent water mol­ecule, are involved. It is inter­esting to note that the dipeptide crystallized as a monohydrate. The water mol­ecule is approximately tetra­hedrally surrounded by four hydrogen bonds. In particular, a hydrogen bond exists between atom O1*W* of the water mol­ecule and atom O3 of the Tyr carboxyl­ate group and atom S1 of Met. The amine N1 group of Met forms hydrogen bonds with atoms O2 and O3 of the Tyr carboxyl­ate group, while the protonated amine N2 group of Met hydrogen bonds not only with atoms O2 and O3 of the Tyr carboxyl­ate group, but also with atom O1*W* of the water mol­ecule. Several weak C—H⋯O hydrogen bonds also occur (Table 1 ▸). The unit cell is shown in Fig. 3 ▸.

## Synthesis and crystallization

The dipeptide l-Met–l-Tyr was obtained commercially (Chemimpex Inter­national, Inc., Wood Dale, IL, USA). To about 100 mg of the dipeptide in a small tube, 2 ml of ethanol was added and mixed throughly on a vortex mixer. Additional solvent was added as required in small increments, while mixing on a vortex mixer and keeping the contents at 60 °C in a water bath. A small amount of water was added at the end to dissolve the peptide completely. The solution was left undisturbed at room temperature for slow evaporation and crystallization.

## Refinement

Crystal data, data collection and structure refinement details are summarized in Table 2 ▸.

## Supplementary Material

Crystal structure: contains datablock(s) I, global. DOI: 10.1107/S2414314623005515/hb4434sup1.cif

Structure factors: contains datablock(s) I. DOI: 10.1107/S2414314623005515/hb4434Isup2.hkl

Supporting information file. DOI: 10.1107/S2414314623005515/hb4434Isup3.cml

CCDC reference: 2260065

Additional supporting information:  crystallographic information; 3D view; checkCIF report

## Figures and Tables

**Table 1:** Hydrogen-bond geometry (Å, °)

*D*—H⋯*A*	*D*—H	H⋯*A*	*D*⋯*A*	*D*—H⋯*A*
N1—H1*N*⋯O2^i^	0.97 (3)	1.95 (3)	2.863 (2)	156 (2)
N2—H21*N*⋯O1*W* ^ii^	0.91 (3)	2.01 (3)	2.805 (2)	145 (2)
N2—H22*N*⋯O3^iii^	0.95 (3)	1.91 (3)	2.827 (2)	162 (2)
N2—H23*N*⋯O2^iv^	0.95 (3)	1.80 (3)	2.743 (2)	171 (2)
C6—H6⋯O1*W*	0.95	2.61	3.290 (3)	129
C11—H11⋯O1^v^	1.00	2.66	3.359 (2)	127
C11—H11⋯O4^i^	1.00	2.49	3.108 (2)	119
C12—H12*A*⋯O2^i^	0.99	2.65	3.440 (2)	137
C13—H13*B*⋯O2^iv^	0.99	2.53	3.472 (2)	159
O1*W*—H1*W*⋯S1^vi^	0.85 (3)	2.54 (3)	3.3674 (16)	165 (3)
O1*W*—H2*W*⋯O3^vii^	0.90 (3)	1.82 (3)	2.716 (2)	179 (3)

Symmetry codes: (i) 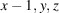 ; (ii) 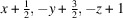 ; (iii) 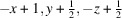 ; (iv) 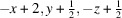 ; (v) 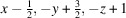 ; (vi) 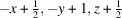 ; (vii) 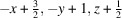 .

**Table 2:** Experimental details

Crystal data	
Chemical formula	C_14_H_20_N_2_O_4_S·H_2_O
*M*_r_	330.39
Crystal system, space group	Orthorhombic, *P*2_1_2_1_2_1_
Temperature (K)	100
*a*, *b*, *c* (Å)	5.4826 (3), 12.4971 (7), 23.5451 (13)
*V* (Å^3^)	1613.23 (15)
*Z*	4
Radiation type	Cu *K*α
μ (mm^−1^)	2.01
Crystal size (mm)	0.29 × 0.08 × 0.02

Data collection	
Diffractometer	Bruker Kappa APEXII DUO CCD
Absorption correction	Multi-scan (*SADABS*; Krause *et al.*, 2015 ▸)
*T*_min_, *T*_max_	0.777, 0.961
No. of measured, independent and observed [*I* > 2σ(*I*)] reflections	14990, 3002, 2854
*R*_int_	0.040
(sin θ/λ)_max_ (Å^−1^)	0.607

Refinement	
*R*[*F* ^2^ > 2σ(*F* ^2^)], *wR*(*F* ^2^), *S*	0.025, 0.064, 1.06
No. of reflections	3002
No. of parameters	221
H-atom treatment	H atoms treated by a mixture of independent and constrained refinement
Δρ_max_, Δρ_min_ (e Å^−3^)	0.22, −0.18
Absolute structure	Flack *x* determined using 1154 quotients [(*I* ^+^) − (*I* ^−^)]/[(*I* ^+^) + (*I* ^−^)] (Parsons *et al.*, 2013 ▸)
Absolute structure parameter	0.031 (7)

Computer programs: *APEX2* and *SAINT* (Bruker, 2016 ▸), *SHELXS97* (Sheldrick, 2008 ▸), *SHELXL2014* (Sheldrick, 2015 ▸), *Mercury* (Macrae *et al.*, 2020 ▸), *ORTEP-3 for Windows* (Farrugia, 2012 ▸), and *publCIF* (Westrip, 2010 ▸).

## full crystallographic data

### Crystal data

**Table:**

C_14_H_20_N_2_O_4_S·H_2_O	*D*_x_ = 1.360 Mg m^−^^3^
*M**_r_* = 330.39	Cu *K*α radiation, λ = 1.54184 Å
Orthorhombic, *P*2_1_2_1_2_1_	Cell parameters from 7969 reflections
*a* = 5.4826 (3) Å	θ = 3.8–69.4°
*b* = 12.4971 (7) Å	µ = 2.01 mm^−^^1^
*c* = 23.5451 (13) Å	*T* = 100 K
*V* = 1613.23 (15) Å^3^	Lath, colourless
*Z* = 4	0.29 × 0.08 × 0.02 mm
*F*(000) = 704	

### Data collection

**Table:**

Bruker Kappa APEXII DUO CCD diffractometer	3002 independent reflections
Radiation source: IµS microfocus	2854 reflections with *I* > 2σ(*I*)
QUAZAR multilayer optics monochromator	*R*_int_ = 0.040
φ and ω scans	θ_max_ = 69.4°, θ_min_ = 4.0°
Absorption correction: multi-scan (SADABS; Krause *et al.*, 2015)	*h* = −4→6
*T*_min_ = 0.777, *T*_max_ = 0.961	*k* = −14→15
14990 measured reflections	*l* = −28→28

### Refinement

**Table:**

Refinement on *F*^2^	Hydrogen site location: mixed
Least-squares matrix: full	H atoms treated by a mixture of independent and constrained refinement
*R*[*F*^2^ > 2σ(*F*^2^)] = 0.025	*w* = 1/[σ^2^(*F*_o_^2^) + (0.033*P*)^2^ + 0.2455*P*] where *P* = (*F*_o_^2^ + 2*F*_c_^2^)/3
*wR*(*F*^2^) = 0.064	(Δ/σ)_max_ = 0.001
*S* = 1.06	Δρ_max_ = 0.22 e Å^−^^3^
3002 reflections	Δρ_min_ = −0.18 e Å^−^^3^
221 parameters	Absolute structure: Flack *x* determined using 1154 quotients [(I+)-(I-)]/[(I+)+(I-)] (Parsons *et al.*, 2013)
0 restraints	Absolute structure parameter: 0.031 (7)
Primary atom site location: structure-invariant direct methods	

### Special details

**Table:**

Geometry. All esds (except the esd in the dihedral angle between two l.s. planes) are estimated using the full covariance matrix. The cell esds are taken into account individually in the estimation of esds in distances, angles and torsion angles; correlations between esds in cell parameters are only used when they are defined by crystal symmetry. An approximate (isotropic) treatment of cell esds is used for estimating esds involving l.s. planes.
Refinement. All H atoms were located in difference maps and those on C atoms were treated thereafter as riding in geometrically idealized positions, with C—H = 0.95 Å for phenyl, 0.99 Å for CH_2_ and 0.98 Å for methyl H atoms. The coordinates of the N—H and O—H H atoms were refined. The *U*_iso_(H) values were assigned as 1.5*U*_eq_ of the attached atom methyl, OH, and ammonium H atoms, and as 1.2*U*_eq_ otherwise.

### Fractional atomic coordinates and isotropic or equivalent isotropic displacement parameters (Å^2^)

**Table:**

	*x*	*y*	*z*	*U*_iso_*/*U*_eq_	
S1	0.45672 (10)	0.72942 (4)	0.10139 (2)	0.01932 (13)	
O1	0.6522 (3)	0.64462 (13)	0.60560 (7)	0.0234 (3)	
H1OH	0.526 (6)	0.613 (2)	0.6171 (13)	0.035*	
O2	1.2758 (2)	0.52566 (10)	0.29568 (6)	0.0150 (3)	
O3	0.9501 (3)	0.54361 (10)	0.23911 (6)	0.0152 (3)	
O4	0.8826 (2)	0.79919 (11)	0.28382 (6)	0.0159 (3)	
N1	0.6958 (3)	0.65289 (13)	0.32141 (7)	0.0131 (3)	
H1N	0.543 (5)	0.6144 (19)	0.3235 (10)	0.016*	
N2	0.4517 (3)	0.90575 (13)	0.27842 (7)	0.0143 (3)	
H21N	0.507 (5)	0.927 (2)	0.3129 (12)	0.022*	
H22N	0.298 (5)	0.938 (2)	0.2718 (11)	0.022*	
H23N	0.557 (5)	0.941 (2)	0.2526 (11)	0.022*	
C1	0.6961 (4)	0.61434 (16)	0.55052 (9)	0.0177 (4)	
C2	0.9078 (4)	0.65037 (18)	0.52474 (9)	0.0206 (4)	
H2	1.0162	0.6960	0.5448	0.025*	
C3	0.9612 (4)	0.61967 (16)	0.46948 (9)	0.0188 (4)	
H3	1.1061	0.6452	0.4520	0.023*	
C4	0.8066 (4)	0.55226 (16)	0.43912 (8)	0.0151 (4)	
C5	0.5910 (4)	0.52002 (16)	0.46491 (9)	0.0183 (4)	
H5	0.4796	0.4765	0.4444	0.022*	
C6	0.5353 (4)	0.55030 (17)	0.52031 (9)	0.0193 (4)	
H6	0.3874	0.5272	0.5374	0.023*	
C7	0.8751 (4)	0.51252 (15)	0.38059 (9)	0.0153 (4)	
H7A	0.7422	0.4661	0.3663	0.018*	
H7B	1.0237	0.4679	0.3838	0.018*	
C8	0.9225 (4)	0.60106 (15)	0.33686 (8)	0.0127 (4)	
H8	1.0308	0.6558	0.3549	0.015*	
C9	1.0589 (4)	0.55304 (14)	0.28579 (8)	0.0127 (4)	
C10	0.6979 (3)	0.74683 (14)	0.29371 (8)	0.0117 (4)	
C11	0.4474 (4)	0.78691 (14)	0.27481 (8)	0.0136 (4)	
H11	0.3216	0.7592	0.3018	0.016*	
C12	0.3816 (3)	0.75003 (16)	0.21469 (8)	0.0156 (4)	
H12A	0.3336	0.6737	0.2163	0.019*	
H12B	0.2376	0.7912	0.2017	0.019*	
C13	0.5835 (4)	0.76236 (17)	0.17061 (8)	0.0171 (4)	
H13A	0.7207	0.7137	0.1796	0.020*	
H13B	0.6454	0.8368	0.1706	0.020*	
C14	0.7332 (5)	0.7195 (2)	0.06034 (11)	0.0367 (6)	
H14A	0.8149	0.7893	0.0596	0.055*	
H14B	0.6935	0.6976	0.0215	0.055*	
H14C	0.8415	0.6664	0.0776	0.055*	
O1W	0.2853 (3)	0.50422 (12)	0.64496 (7)	0.0195 (3)	
H1W	0.240 (5)	0.447 (2)	0.6281 (13)	0.029*	
H2W	0.372 (6)	0.487 (2)	0.6762 (14)	0.029*	

### Atomic displacement parameters (Å^2^)

**Table:**

	*U*^11^	*U*^22^	*U*^33^	*U*^12^	*U*^13^	*U*^23^
S1	0.0240 (2)	0.0196 (2)	0.0143 (2)	−0.0005 (2)	−0.0008 (2)	−0.00063 (19)
O1	0.0273 (8)	0.0285 (8)	0.0144 (7)	−0.0058 (7)	0.0056 (7)	−0.0028 (6)
O2	0.0133 (7)	0.0133 (6)	0.0185 (7)	0.0011 (5)	0.0002 (6)	−0.0008 (6)
O3	0.0163 (7)	0.0156 (6)	0.0136 (6)	−0.0017 (6)	−0.0017 (6)	−0.0014 (5)
O4	0.0127 (7)	0.0136 (7)	0.0214 (7)	−0.0007 (5)	0.0007 (5)	0.0018 (5)
N1	0.0116 (8)	0.0115 (7)	0.0161 (8)	−0.0002 (7)	0.0001 (6)	0.0010 (6)
N2	0.0142 (8)	0.0134 (8)	0.0154 (8)	0.0014 (7)	0.0003 (7)	0.0010 (7)
C1	0.0229 (11)	0.0181 (10)	0.0123 (10)	0.0032 (8)	0.0012 (8)	0.0002 (8)
C2	0.0220 (11)	0.0228 (10)	0.0171 (10)	−0.0029 (9)	−0.0004 (9)	−0.0016 (8)
C3	0.0165 (9)	0.0229 (10)	0.0169 (10)	−0.0017 (9)	0.0018 (9)	0.0018 (8)
C4	0.0181 (10)	0.0133 (9)	0.0139 (10)	0.0042 (8)	0.0005 (8)	0.0038 (8)
C5	0.0210 (11)	0.0179 (9)	0.0160 (10)	−0.0031 (8)	−0.0017 (8)	0.0009 (8)
C6	0.0180 (10)	0.0226 (10)	0.0175 (10)	−0.0008 (9)	0.0023 (9)	0.0021 (8)
C7	0.0181 (10)	0.0120 (9)	0.0158 (10)	0.0015 (8)	0.0012 (8)	0.0017 (7)
C8	0.0141 (10)	0.0117 (8)	0.0124 (9)	0.0009 (8)	−0.0007 (8)	−0.0006 (7)
C9	0.0135 (9)	0.0076 (8)	0.0169 (10)	−0.0029 (8)	0.0000 (8)	0.0010 (7)
C10	0.0136 (9)	0.0109 (9)	0.0105 (9)	0.0012 (7)	0.0015 (7)	−0.0018 (7)
C11	0.0141 (9)	0.0122 (9)	0.0145 (9)	0.0001 (8)	0.0010 (8)	0.0019 (7)
C12	0.0149 (9)	0.0162 (9)	0.0158 (10)	−0.0023 (7)	−0.0025 (7)	0.0014 (8)
C13	0.0186 (10)	0.0177 (9)	0.0149 (10)	−0.0014 (8)	0.0012 (8)	−0.0016 (7)
C14	0.0377 (14)	0.0502 (16)	0.0222 (13)	0.0036 (13)	0.0064 (10)	−0.0097 (12)
O1W	0.0249 (8)	0.0188 (7)	0.0147 (7)	−0.0028 (6)	−0.0006 (6)	−0.0001 (6)

### Geometric parameters (Å, º)

**Table:**

S1—C14	1.802 (3)	C5—C6	1.392 (3)
S1—C13	1.819 (2)	C5—H5	0.9500
O1—C1	1.372 (3)	C6—H6	0.9500
O1—H1OH	0.84 (3)	C7—C8	1.534 (3)
O2—C9	1.259 (3)	C7—H7A	0.9900
O3—C9	1.256 (2)	C7—H7B	0.9900
O4—C10	1.228 (2)	C8—C9	1.538 (3)
N1—C10	1.343 (2)	C8—H8	1.0000
N1—C8	1.448 (3)	C10—C11	1.528 (3)
N1—H1N	0.97 (3)	C11—C12	1.532 (3)
N2—C11	1.488 (2)	C11—H11	1.0000
N2—H21N	0.91 (3)	C12—C13	1.525 (3)
N2—H22N	0.95 (3)	C12—H12A	0.9900
N2—H23N	0.95 (3)	C12—H12B	0.9900
C1—C2	1.385 (3)	C13—H13A	0.9900
C1—C6	1.387 (3)	C13—H13B	0.9900
C2—C3	1.388 (3)	C14—H14A	0.9800
C2—H2	0.9500	C14—H14B	0.9800
C3—C4	1.392 (3)	C14—H14C	0.9800
C3—H3	0.9500	O1W—H1W	0.85 (3)
C4—C5	1.388 (3)	O1W—H2W	0.90 (3)
C4—C7	1.512 (3)		
			
C14—S1—C13	100.05 (11)	N1—C8—C9	113.30 (16)
C1—O1—H1OH	109 (2)	C7—C8—C9	109.01 (15)
C10—N1—C8	120.38 (16)	N1—C8—H8	108.0
C10—N1—H1N	117.9 (14)	C7—C8—H8	108.0
C8—N1—H1N	120.5 (14)	C9—C8—H8	108.0
C11—N2—H21N	110.3 (16)	O3—C9—O2	125.79 (19)
C11—N2—H22N	113.9 (16)	O3—C9—C8	119.33 (17)
H21N—N2—H22N	109 (2)	O2—C9—C8	114.87 (17)
C11—N2—H23N	116.0 (16)	O4—C10—N1	124.39 (18)
H21N—N2—H23N	104 (2)	O4—C10—C11	120.75 (16)
H22N—N2—H23N	104 (2)	N1—C10—C11	114.86 (16)
O1—C1—C2	118.13 (19)	N2—C11—C10	107.23 (16)
O1—C1—C6	122.15 (19)	N2—C11—C12	110.91 (15)
C2—C1—C6	119.7 (2)	C10—C11—C12	112.47 (16)
C1—C2—C3	119.8 (2)	N2—C11—H11	108.7
C1—C2—H2	120.1	C10—C11—H11	108.7
C3—C2—H2	120.1	C12—C11—H11	108.7
C2—C3—C4	121.3 (2)	C13—C12—C11	115.31 (16)
C2—C3—H3	119.3	C13—C12—H12A	108.4
C4—C3—H3	119.3	C11—C12—H12A	108.4
C5—C4—C3	117.98 (19)	C13—C12—H12B	108.4
C5—C4—C7	120.98 (19)	C11—C12—H12B	108.4
C3—C4—C7	121.01 (19)	H12A—C12—H12B	107.5
C4—C5—C6	121.2 (2)	C12—C13—S1	108.02 (14)
C4—C5—H5	119.4	C12—C13—H13A	110.1
C6—C5—H5	119.4	S1—C13—H13A	110.1
C1—C6—C5	119.9 (2)	C12—C13—H13B	110.1
C1—C6—H6	120.1	S1—C13—H13B	110.1
C5—C6—H6	120.1	H13A—C13—H13B	108.4
C4—C7—C8	114.64 (16)	S1—C14—H14A	109.5
C4—C7—H7A	108.6	S1—C14—H14B	109.5
C8—C7—H7A	108.6	H14A—C14—H14B	109.5
C4—C7—H7B	108.6	S1—C14—H14C	109.5
C8—C7—H7B	108.6	H14A—C14—H14C	109.5
H7A—C7—H7B	107.6	H14B—C14—H14C	109.5
N1—C8—C7	110.24 (16)	H1W—O1W—H2W	110 (3)
			
O1—C1—C2—C3	178.66 (19)	C4—C7—C8—C9	163.90 (16)
C6—C1—C2—C3	−1.9 (3)	N1—C8—C9—O3	−13.3 (2)
C1—C2—C3—C4	−0.5 (3)	C7—C8—C9—O3	109.8 (2)
C2—C3—C4—C5	2.7 (3)	N1—C8—C9—O2	167.13 (16)
C2—C3—C4—C7	−175.37 (19)	C7—C8—C9—O2	−69.7 (2)
C3—C4—C5—C6	−2.7 (3)	C8—N1—C10—O4	−6.3 (3)
C7—C4—C5—C6	175.42 (19)	C8—N1—C10—C11	174.12 (16)
O1—C1—C6—C5	−178.65 (19)	O4—C10—C11—N2	−33.4 (2)
C2—C1—C6—C5	1.9 (3)	N1—C10—C11—N2	146.24 (16)
C4—C5—C6—C1	0.4 (3)	O4—C10—C11—C12	88.8 (2)
C5—C4—C7—C8	123.7 (2)	N1—C10—C11—C12	−91.57 (19)
C3—C4—C7—C8	−58.3 (3)	N2—C11—C12—C13	74.5 (2)
C10—N1—C8—C7	165.36 (17)	C10—C11—C12—C13	−45.6 (2)
C10—N1—C8—C9	−72.2 (2)	C11—C12—C13—S1	−173.31 (13)
C4—C7—C8—N1	−71.1 (2)	C14—S1—C13—C12	−167.63 (16)

### Hydrogen-bond geometry (Å, º)

**Table:**

*D*—H···*A*	*D*—H	H···*A*	*D*···*A*	*D*—H···*A*
N1—H1*N*···O2^i^	0.97 (3)	1.95 (3)	2.863 (2)	156 (2)
N2—H21*N*···O1*W*^ii^	0.91 (3)	2.01 (3)	2.805 (2)	145 (2)
N2—H22*N*···O3^iii^	0.95 (3)	1.91 (3)	2.827 (2)	162 (2)
N2—H23*N*···O2^iv^	0.95 (3)	1.80 (3)	2.743 (2)	171 (2)
C6—H6···O1*W*	0.95	2.61	3.290 (3)	129
C11—H11···O1^v^	1.00	2.66	3.359 (2)	127
C11—H11···O4^i^	1.00	2.49	3.108 (2)	119
C12—H12*A*···O2^i^	0.99	2.65	3.440 (2)	137
C13—H13*B*···O2^iv^	0.99	2.53	3.472 (2)	159
O1*W*—H1*W*···S1^vi^	0.85 (3)	2.54 (3)	3.3674 (16)	165 (3)
O1*W*—H2*W*···O3^vii^	0.90 (3)	1.82 (3)	2.716 (2)	179 (3)

Symmetry codes: (i) *x*−1, *y*, *z*; (ii) *x*+1/2, −*y*+3/2, −*z*+1; (iii) −*x*+1, *y*+1/2, −*z*+1/2; (iv) −*x*+2, *y*+1/2, −*z*+1/2; (v) *x*−1/2, −*y*+3/2, −*z*+1; (vi) −*x*+1/2, −*y*+1, *z*+1/2; (vii) −*x*+3/2, −*y*+1, *z*+1/2.
